# Can mini PCNL achieve the same results as RIRS? The initial single center experience

**DOI:** 10.1016/j.amsu.2021.102632

**Published:** 2021-07-31

**Authors:** Shawqi George Ghazala, Sarbast Mohammed Saeed Ahmed, Ayad Ahmad Mohammed

**Affiliations:** a Department of Surgry, Hawler Medical University, Erbil, Kurdistan Region, Iraq; b Department of Surgery, Duhok Directorate General of Health, DUHOK, Kurdistan Region, Iraq; c Department of Surgery, College of Medicine, University of Duhok, DUHOK, Kurdistan Region, Iraq

**Keywords:** Mini-PCNL, RIRS, Renal stones, JJ stent, Urolithiasis, Laser for renal stones

## Abstract

**Background:**

Urolithiasis is a prevalent disease worldwide with high recurrence rate, minimally invasive interventions have largely replaced open ones, namely PCNL and RIRS. Miniaturization, optical improvements, and modern laser types made these procedures safe and effective in the management of single renal stones.

**Aim of the study**: Is to compare the effectiveness of mini PCNL with RIRS in the treatment of single renal stone of ≤25 mm.

**Patients and methods:**

This prospective study that included 60 patients with single renal stones of ≤25 mm and were treated by either mini PCNL (group A) or RIRS (group B). The study was performed during the period from October 2020 to April 2021.

**Results:**

The mean operative time RIRS group was 43.6 ± 10.493, while for miniPCNL it was 36.6 ± 7.035 (P = 0.004). The stone free rate in RIRS and miniPCNL group was 70% and 90% respectively (P = 0.053). The need for JJ stent was higher in RIRS compared to miniPCNL group (70% vs. 40%) respectively (P = 0.02). The duration of hospital stay in miniPCNL was 38.2 h compared to 16.7 h for RIRS group (p = 0.0001). The rate of postoperative hemoglobin drop was higher in MiniPCNL compared to RIRS (P = 0.0001). There was no significant difference regarding complication rates between both groups.

**Conclusion:**

Mini-PCNL FOR the treatment of renal stones sized ≤25 mm has high stone free rate, shorter operative time, less requirement for JJ stent and near similar post-operative pain and complications compared to RIRS.

## Highlights

• Urolithiasis is a prevalent disease worldwide with high recurrence rate**.**
• Minimally invasive interventions have largely replaced open ones.
• Advancement of the technology have improved the management of stones.

## 1 Introduction

Urinary tract stones commonly affect human and the incidence of renal stones has been increased in over the last decades. They are a very common cause of morbidity worldwide. The lifetime risk for the development of stone development is around 5–10%. Renal stones may be recurrent in many patients, the lifetime recurrence is reported to be up to 50% [1,2].

Advancement of the technology have improved the approach to the management of the stones. The minimally invasive techniques like extracorporeal shock wave lithotripsy (ESWL), retrograde intra-renal surgery (RIRS), percutaneous nephrolithotomy (PCNL) and laparoscopic ureterolithotomy, have largely replaced the open surgical techniques [2].

Percutaneous nephrolithotomy (PCNL) was first practiced around 40 years ago, since that time it has underwent many modifications, innovations, and minimization. The new modifications greatly focused on the achievement of delivering greater stone clearance, and reducing morbidity, surgical time and the duration of hospital stay in the other hand [3].

The complications include hemorrhage and organ trauma which occur as the result of creation of the tract and dilation in the standard PCNL, minimizing the size of the instruments as well as the introduction of laser technology and improvement of the optical systems have resulted in reduction in the rate of the complications and the invention of new techniques. Recently an increasing number of articles have been done about the efficacy of the mini-PCNL and comparing it to the standard technique [3,4].

Miniaturization in PCNL has been similar in retrograde intra-renal surgery (RIRS), with smaller caliber ureterorenoscopes with larger and more durable working channels. Ureterorenoscopic miniaturization, better visualization, improved deflective capability and the usage of ureteric access sheaths indicates that a wider range of renal stones are now being tackled ureteroscopically [[5], [6], [7]].

RIRS is done through natural orifices. It can decrease the duration of the hospital stay and the risk of hemorrhage. Studies indicated that although the safety is more guaranteed, RIRS may not be very effective in the management of stones larger than 2.0 cm [8].

With larger and more complex stones being treated ureteroscopically and smaller stones being managed by miniaturized PCNL tracts, there is an imperative requirement need to provide an evidence as to the relative outcomes and indications of these two procedures [8].

The aim of the study is to compare the effectiveness of mini PCNL with RIRS in the treatment of single renal stone of ≤25 mm.

### 1.1 Patients and methods

This is a prospective study that included 60 patients from two academic centers in Erbil/Iraq. Patients were enrolled from the period of October 2020 to April 2020. Patients are divided in to groups labeled A and B respectively. Group A include 30 patients who underwent mini PCNL and group B include 30 patients underwent RIRS.

**Inclusion criteria:** patients with single renal stone of all types and sized ≤25 mm, who aged ≥23 years old, and those with normal renal functions were included.

**Exclusion criteria:** Patients with multiple renal stones, staghorn stones, those who were ≤23 years old**,** uncorrected coagulopathy, active UTI**,** and those with musculoskeletal abnormalities were excluded.

**Preoperative preparation:** Preoperatively medical history taken and physical examination performed from all patients. Laboratory studies; urine analysis, complete count, renal function test, serum electrolytes, bleeding tendency profile, fasting blood sugar and virology profile done for all patients. Preoperative ECG, echocardiography and chest X-ray done for selected patients. Imaging studies inform of ultrasonography, intravenous pyelography including (KUB) or CT-urography done to locate the site, size, and laterality of stone and anatomy of the pelvicalyceal system.

**Operation preparation:** Preoperative medical written consent taken from patients and prophylactic antibiotic inform of third generation cephalosporine 1 g iv administered at the induction of anesthesia. The choice of the surgical method was decided by the surgical team discussion with patient's expectation and outcome.

**Mini-PCNL technique:** After induction of general anesthesia, patients were placed in the dorsal lithotomy position, urethral cystoscope was done with a 20 Fr rigid cystoscope and a hydrophilic guide wire was inserted into the relevant ureter. Then a 5 Fr open-ended ureter catheter was advanced and a16Fr foleys catheter was fixed. Then the patient was turned to prone position and 50% diluted nonionic contrast material pushed through the ureteric catheter under fluoroscopy and all calices were allowed to fill with radio-opaque material. Under C-arm fluoroscopy 18-gauge needle (shiba) inserted to the targeted calyx (usually lower calix) a guide wire was passed through to the pelvicalyceal system then Alken metal dilatators were inserted in the kidney over the guide wire. Then a 22 Fr operating sheath was inserted above the dilators, and the lower calyx was entered with 18 Fr nephroscope and the pelvicalyceal system and stones were evaluated then either stones will be taken without fragmentation or if needed stones were fragmented with a pneumatic lithotripter. Otherwise, large stones were fragmented and these were removed. At the end of surgery, fluoroscopy and nephroscope were used to confirm there was complete stone clearance inside the kidney. A20 Fr nelaton tube was left in selected patients as nephrostomy tube. Additionally, JJ stent insertion was optional depending on local tissue trauma and presence the amount of gravel's left behind.

**RIRS technique:** After induction of general anesthesia, the patient put on dorsal lithotomy position, and rigid URS performed in the relevant side, a 0.035 Fr ureteric guide wire put under vision. A 12 Fr ureteric access sheath fixed over the guide wire. 7.5 Fr flexible ureteroscope (Karl Storz, Tuttlingen, Germany) introduced through the access sheath to the collecting system. After localization of the stone, fragmentation of stones performed by Holmium YAG-laser (LITHO-QUANTA SYSTEM Holmium laser system) lithotripter with 200 μm fiber for lithotripsy. The laser machine was adjusted according to the type of stones treatment either fragmentation or dusting modes. Dusting mode use high frequency (10–20 Hz) low energy (0,5 J) energy while fragmentation mode of low frequency (5–10 Hz) and high energy (2–3 J). Stone fragments were removed by nitinol stone basket. At the end of procedure JJ stent was put depending on local criteria and operating time. All calices were checked for residual stone by flexible URS and fluoroscopy for radio-opaque stones intraoperatively.

**Follow up:** During hospital stay, patients received iv fluid, iv antibiotic and analgesia. Follow up of patients done regarding stone free rate by imaging studies {Ultrasonography, plain abdominal x-ray (KUB)} [1] and non-contrast CT after one month and JJ stent removed after confirmation of stone clearance.

In accordance to the World Medical Association's Declaration of Helsinki 2013, the work of this article is registered in the Research Registry, and the unique identifying number is: researchregistry 6891.

The link to the registration page is:

The work of this article has been reported in line with the STROCSS criteria [9].

## 2 Results

The mean age of the patients in the RIRS group was 45.07 ± 13.329, while for the miniPCNL group it was 44.73 ± 13.292, and males constituted the majority of the patients in both groups; 66.7% for the RIRS group and 63.3% for the other one. About 70% of the stones were in the right site for the RIRS group, while right side stones constituted 50% for the other group. Table 1.

**Table 1:** Showing the patient's and stone charecteristics.

Category	MiniPCNL	RIRS
Age (M; SD)	44.73 (13.292)	45.07 (13.329)
Sex Male Female	19 (63.3%) 11 (36.7%)	20 (66.7%) 10 (33.3%)
Laterality Right Left	15 (50.0%) 15 (50.0%)	21 (70.0%) 9 (30.0%)
Stone size in mm (M; SD)	19.8 (3.517)	16.6 (4.415)

**Fig. 1:**
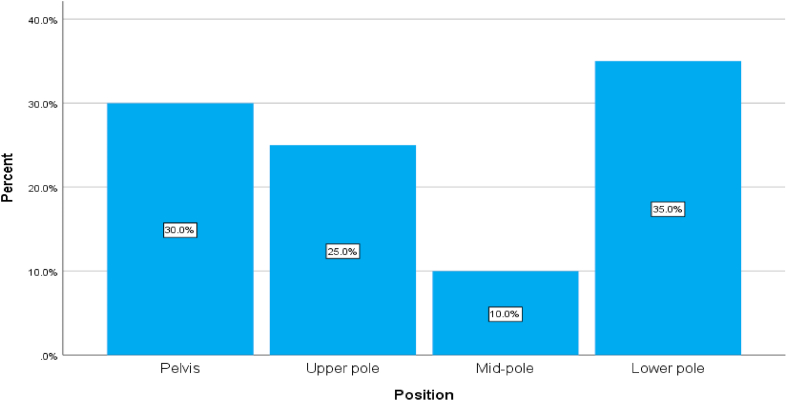
A simple bar chart showing the position of the stones in both groups.

35%,30% of the stones were located in the lower pole and pelvis respectively. Fig. 1.

**Fig. 2:**
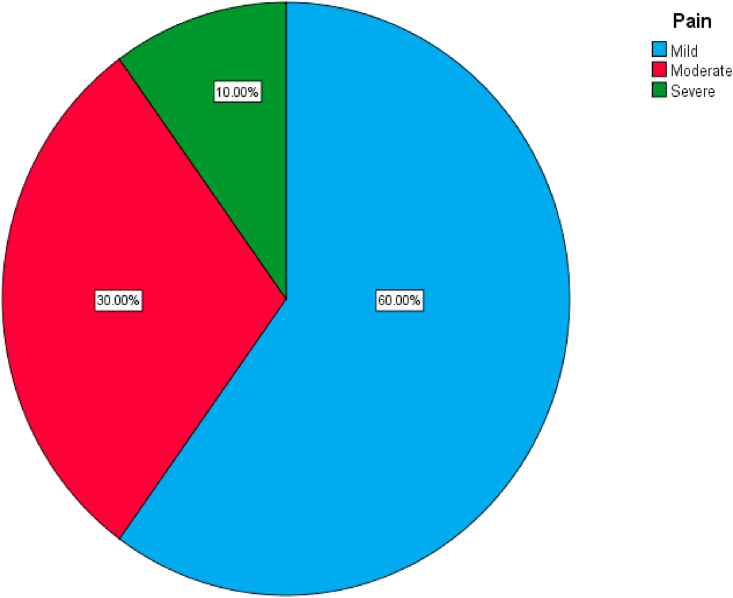
A simple pie chart showing the degree of the pain of patients in the MiniPCNL group after surgery.

**Fig. 3:**
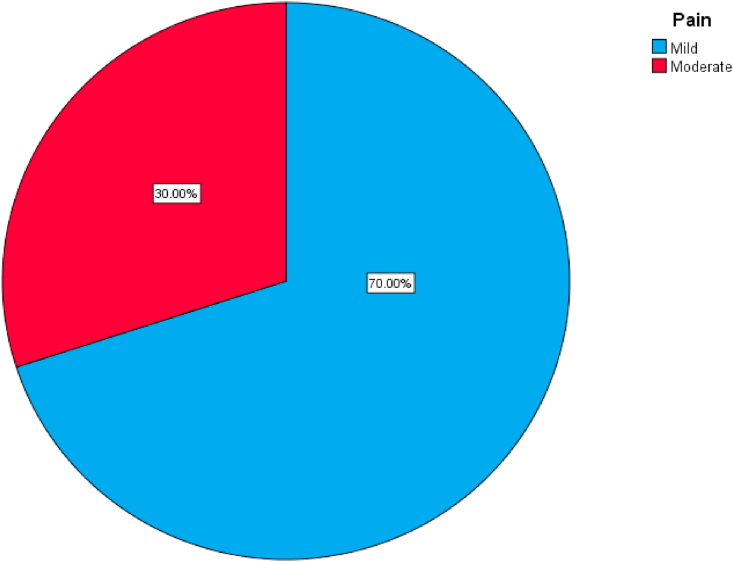
A simple pie chart showing the degree of the pain of patients in the RIRS group after surgery.

The mean operative time for patients in the RIRS group was 43.6 ± 10.493, while for those in the miniPCNL was 36.6 ± 7.035. The stone free rate in RIRS and miniPCNL group was 70% and 90% respectively. The need for JJ stent insertion was higher in RIRS group compared to miniPCNL (70% vs. 40%) for each variable. Ten percent of patients with miniPNL showed a drop in the renal function after surgery. The duration of hospital stay in minPCNL was 38.2 h compared to 16.7 h for RIRS group. The majority of patients had mild pain after operation. Table 2, Fig. 2, Fig. 3.

**Table 2:** Showing some of the operation details and the post operative follow up.

Category	MiniPCNL	RIRS
Operative time in minutes (M; SD)	36.6 (7.035)	43.6 (10.493)
Stone free? No Yes	3 (10.0%) 27 (90.0%)	9 (30.0%) 21 (70.0%)
**Need for JJ stent** No Yes	18 (60.0%) 12 (40.0%)	9 (30.0%) 21 (70.0%)
Hb drop (M; SD)	0.89 (0.267)	0.33 (0.1512)
**RFT** Normal Increase	27 (90.0%) 3 (10.0%)	30 (100.0%) 0 (0.0%)
Hospital Stay in hours (M:SD)	38.2 (8.616)	16.7 (4.772)

Comparison was done between both groups of intervention, the correlation was done using the Pearson's Chi square test and the Fischer's exact test, there was a significant difference between both groups regarding the need for the JJ stents and hematuria. Table 3.

**Table 3:** Showing the comparisons of the categorial variables between both groups.

Categories	Type of operation		Sig. (2-sided)
	Mini PCNL (n = 30)	RIRS (n = 30)	
Gender Male Female	19 (63.3%) 11 (36.7%)	20 (66.7%) 10 (33.3%)	0.787*
Laterality Right Left	15 (50.0%) 15 (50.0%)	21 (70.0%) 9 (30.0%)	0.114*
Position Pelvis Upper pole Mid-pole Lower pole	9 (30.0%) 6 (20.0%) 3 (10.0%) 12 (40.0%)	9 (30.0%) 9 (30.0%) 3 (10.0%) 9 (30.0%)	0.797**
Stone free? No Yes	3 (10.0%) 27 (90.0%)	9 (30.0%) 21 (70.0%)	0.053*
Need for JJ stent No Yes	18 (60.0%) 12 (40.0%)	9 (30.0%) 21 (70.0%)	0.020*
Pain Mild Moderate Severe	18 (60.0%) 9 (30.0%) 3 (10.0%)	21 (70.0%) 9 (30.0%) 0 (0.0%)	0.355**
RFT Normal Increase	27 (90.0%) 3 (10.0%)	30 (100.0%) 0 (0.0%)	0.237**
Complications No Yes	6 (20.0%) 24 (80.0%)	9 (30.0%) 21 (70.0%)	0.371*
Bleeding No Yes	27 (90.0%) 3 (10.0%)	30 (100.0%) 0 (0.0%)	0.237**
Hematuria No Yes	9 (30.0%) 21 (70.0%)	18 (60.0%) 12 (40.0%)	0.020*
Post-op fever No Yes	24 (80.0%) 6 (20.0%)	21 (70.0%) 9 (30.0%)	0.371*

*Pearson's Chi square test, ** Fischer Exact test.

The independent *t*-test was performed to detect any significant differences between both groups regarding the numerical variables, there was a significant difference between both groups in regard to the stone size, operation time, drop in the HB, and the length of the hospital stay. Table 4.

**Table 4:** Showing the comparisons of the numerical variables between both groups.

Categories	Mean Difference	Std. Error Difference	95% Confidence Interval of the Difference		Sig. (2-tailed)
			Lower	Upper	
Age	.333	3.437	−6.546	.923	.923
Stone size in mm	−3.200	1.031	−5.263	.003	**.003**
Operative time in minutes	7.000	2.307	2.383	.004	**.004**
Hb drop	-.5600	.0560	-.6721	.0001	**.0001**
Hospital Stay	−21.500	1.798	−25.099	.0001	**.0001**

## 3 Discussion

Mini-PCNL is effective with less blood loss in small and medium size stone. Even a complex stone burden may be amenable to mini-PCNL. The comparisons were not adjusted for different technical details like puncture guidance, type of dilators, tract size, and the type of lithotripsy used. The preoperative stone size and the stone location constitute important parameters, for choosing the best treatment [10,11].

Percutaneous nephrolithotomy must be obtained under anesthesia. A small skin incision is made and a nephroscope is progressed into the renal pelvicalyceal system to check the stones. Stones are fragmented by using either laser or pneumatic lithotripsy through the nephroscope and then the stone fragments are removed. Finally, a nephrostomy tube is placed for either hemostasis or drainage of the kidney. Retrograde intrarenal surgery is a minimally invasive surgical procedure using flexible ureteroscope entering the urethra through the bladder, the ureter, into the renal pelvicalyceal system. This procedure is a retrograde approach to the intrarenal urine‐collecting part and normally done under an anesthesia. The stones can be seen through the scope, then treated with intracorporeal lithotriptors and removed by using grasping devices. At present, RIRS is commonly used to remove stones from the kidney [[12], [13], [14]].

The mean age of the patients in the RIRS group was 45.07 ± 13.329, while for the miniPCNL group was 44.73 ± 13.292, (P = 0.923), and males constituted the majority of the patients in both groups; 66.7% for the RIRS group and 63.3% for the other group. About 70% of the stones were in the right site for the RIRS group, while right side stones constituted 50% for the other group (P = 0.114). Regarding the stone size, there was a significant differences between both groups (P value 0.003), larger stones are difficult to be managed with RIRS, while the position of the stone showed no any significant correlation between both groups (P value 0.797) [8].

The stone free rate in RIRS and miniPCNL group was 70% and 90% respectively (P = 0.053). The stone free rate in the current study for both miniPCNL and RIRS group is regarded as an acceptable rate when compared to other published articles which showed approximate rates [5,15].

The need for JJ stent insertion was higher in RIRS when compared to miniPCNL group (70% vs. 40%) for each variable (P = 0.020). Ten percent of patients with the miniPCNL showed a mild deterioration in the renal function after surgery that return to normal few days later, and the duration of the hospital stay was higher in patients with miniPCNL. The majority of the patients had mild postoperative pain. The mean operative time for patients in the RIRS group was 43.6 ± 10.493, while for those in the miniPCNL was 36.6 ± 7.035 (P = 0.004) studies have shown average operative times ranging from 40 to 59 min which is comparable to our average of 40.1 min [5].

There was no significant difference regarding the complication rates between both groups (P value 0.237), when comparing the complications separately, there was a significant different regarding the development of hematuria being higher in the Mini PCNL group (P value 0.02). Most studies detected no significant differences in regards to the development of the complications [1,16].

The rate of the Hb drop after surgery was significant between the two groups (P value 0.0001). Other studies showed that the rate of the Hb drop is approximately around 1.2 gm, in our study the rate of the Hb drop was around 0.6 gm/dl in both group collectively but was relatively higher in miniPCNL group which is lower than other studies [5,17,18].

The majority of patients of the RIRS have mild to moderate pain after operation, and none had severe pain, while 10% of patients of the miniPCNL showed severe form of pain and the rest showed mild pain in 60% and moderate pain in 30%. There was no significant difference regarding the pain intensity between both groups (P value 0.355). The mean hospital stay after surgery was 16.7 (4.772) hours for the RIRS groups and 38.2 (8.616) for the miniPCNL group, and the difference was a significant one between both groups (P value 0.0001) being less in the RIRS group [1,19].

The major limitation of our study is a relatively low sample numbers in both groups. The main drawback for RIRS is the high cost of flexible URS equipment and laser lithotripsy.

## 4 Conclusion

Mini-PCNL for the treatment of renal stones sized ≤25 mm has higher stone free rate, shorter operative time, more hematuria, higher Hb drop, longer hospital stays, less requirement for JJ stent and near similar post-operative pain and complications when compared to RIRS.

## Funding

The author is the only source of funding.

## Declaration of competing interest

The authors declare that they have no known competing financial interests or personal relationships that could have appeared to influence the work reported in this paper.

No conflicts of interest present.

No source of funding other than the authors.

NA.

Researchregistry6891.

N/A.

Study design, data collection and analysis, writing and final approval of the manuscript: Dr. Shawqi George Ghazala, Dr Sarbast Mohammed Saeed Ahmed, and Dr Ayad Ahmad Mohammed.

Dr Ayad Ahmad Mohammed.
